# Evaluation of the executive functions and quality of life in a sample of Egyptian male adolescents with substance use disorder: A case-control study

**DOI:** 10.1007/s44192-024-00060-x

**Published:** 2024-03-04

**Authors:** Hassan Mohammed Sonbol, Youmna Sabri, Mohamed Shahda, Eman Abdallah Shouman

**Affiliations:** Department of Psychiatry, Faculty of Medicine-Mansoura University, El-Mansoura, Egypt

**Keywords:** Adolescents, Quality of life, Executive functions, Substance use

## Abstract

**Background:**

Adolescent substance use is a major problem that has serious medical, psychological, and legal consequences later in life. Substance use disorder is closely linked to deficits in executive functions. Impaired executive functions (EFs) have been linked to all stages of the substance use disorder (SUD) life cycle, increasing the likelihood of commencing use, escalating use more quickly, and increasing the likelihood of relapsing following treatment. The current study aimed at evaluating of the executive functions and quality of life in a sample of adolescent Egyptian males with substance use disorder.

**Results:**

A significantly higher mean Trail Making Test-A, B (TMT-A and TMT-B) scores among studied cases than the control group (equals lower executive functions) with a mean score of TMT-A is 74.38 versus 63.2 among controls and for TMT-B; the mean score for control is 97.22 versus 142.04 among cases. A statistically significant difference between the case and control groups on all quality of life scores measuring the following domains: general health and well-being, physical health, psychological health, social interactions, and the environment, also there has been a negative correlation between TMT-A and the environmental domain (r = − 0.279) and TMT-B with the same variable (r = − 0.414).

**Conclusions:**

Substance use disorders are a major health problem among youth. Deficits in executive functions are strongly associated with adolescent substance use. The more affected executive functions are associated with more affected quality of life of these patients.

## Introduction

### Background

The term "substance use" is defined as the harmful or risky use of alcohol, illegal narcotics, and psychotropic substances. Repetitive use of psychoactive substances can result in dependency syndrome, a collection of behavioral, cognitive, and physiological symptoms. Dependency also includes having a strong desire to use the substance, finding it difficult to stop, using it despite the harmful consequences, putting it before other obligations and commitments, developing a tolerance, and going through physical withdrawal [1].

Twenty percent of Egyptian male students in one research reported using drugs, with another 25 percent reporting ongoing drug usage. There were 5.05% of male secondary school students who abused cannabis, 0.84% who abused opiates, 2.72% who abused tranquilizers, 1.79% who abused stimulants, and 2.26% who abused hypnotics. In the most recent National Survey, 9.6% of Egyptians reported ever having taken illegal substances [2].

To successfully negotiate the developmental transition between adolescence and adulthood, adolescents must maneuver this stressful period while learning skills essential for independence. Adolescents of various mammalian species frequently display certain behavioral traits, such as age-related increases in risk-taking and novelty-seeking. These characteristics may support this process. Teenagers may explore novel appetitive reinforcers through drug usage and other risk-taking behaviors as a result of decreased positive incentive values from stimuli, with their relative insensitivity to drugs encouraging proportionally greater per-occasion consumption [3].

Adolescence is a vulnerable time for the emergence of substance use disorders. Recent research in neuropsychology and neuroimaging has shown underlying brain vulnerabilities that play a role in adolescent substance use initiation. Findings indicate that adolescent substance use is predicted with lower performance on inhibition and working memory tests, smaller brain sizes in reward and cognitive control regions, less brain activation during executive functioning tasks, and increased reward responsiveness. Adolescents with a family history of substance use disorder have been found to have worse executive functioning, smaller limbic brain volumes (such as the amygdala), sex-specific hippocampal volume patterns, and a positive correlation between nucleus accumbens volume and family history density [4].

Executive functions (EFs) are a broad concept that incorporates many distinct parts of cognition. Some of the various cognitive aspects that make up EFs include working memory, shifting, planning, problem-solving, and inhibitory control. EF may more directly connect to a person's ability to set goals, prepare, adapt, self-monitor, and make adjustments that are appropriate for the situation. Problems with information processing, systematic action, and daily functioning may result from impaired performance in this area [5].

It is typical for EF to be engaged in risky behavior that has immediate rewards but long-term negative effects, including substance use [6]. Low EFs may be linked to substance use disorder because either the substance use lowers the executive functions [7], or low executive functions may be a risk factor for substance use disorder. In the AUD and SUD populations, early relapse and poor treatment compliance have been associated with deficits in lower-order executive functioning (such as response inhibition) and higher-order executive functioning (such as problem-solving and decision-making) [8].

The World Health Organization defines quality of life (QoL) as a "state of complete physical, mental, and social well-being" that is primarily subjective and prone to change based on individual perceptions, experiences, and expectations. QoL is not only the absence of sickness. The chronic disease paradigm of integrated medical, behavioral, and social assistance is beneficial for patients with substance use disorders since it is recognized as a chronic illness that negatively affects their quality of life. There is evidence that substance abuse is linked to lower quality of life [9]. Substance abuse is linked to reductions in many different areas of QoL, such as physical, social, psychological, labor, scholastic, and financial functioning. In certain circumstances, harm may be done in a particular domain. The quality of life of people with SUDs is negatively impacted in all areas, including their employment, social interactions, interpersonal connections, and physical and mental health [10].

### Research aims

In the current study, we adopted the hypothesis that Substance use disorder is associated with affected executive functions in the adolescent age group, which in turn leads to lower quality of life in these patients. This study aimed to assess some aspects of executive functions and quality of life in male adolescents with substance use disorders.

## Methods

### Study design

A case–control study.

### Setting and duration

The participants in this research were divided into two groups. 50 adolescents who met the Diagnostic and Statistical Manual-5 (DSM-5) Diagnostic Criteria for substance use disorder were the patient group, and another 50 age and gender-matched adolescents with no SUD diagnosis were the control group. Between September 2020 and March 2021, participants were recruited from the outpatient clinic of the psychiatry department's addiction unit at Mansoura University Hospital.

### Participants and procedures

There were 97 adolescents diagnosed clinically as patients with substance use disorders throughout the recruitment process of the research.15 patients declined to participate in the study, while 30 patients did not match the inclusion requirements (either new cases in the intoxication or withdrawal syndrome or illiterate patients). Two of the remaining 52 interviews were conducted but not finished. The database held information on 50 adolescent males in total.

The control group consisted of 50 adolescents of matched age and gender who were free from any neurological or mental conditions at the time of conducting the study. These young people were chosen among those who visited the Mansoura University Hospital’s outpatient clinics for reasons other than psychiatric problems.

Each participant was examined clinically using a structured sheet created for research, including sociodemographic and clinical characteristics, and psychometric tools of assessment, and to exclude the psychiatric disorders in the case and the control groups we utilized MINI-KID.

#### Inclusion criteria of the case group

Meeting the DSM-5 Diagnostic Criteria of substance use disorder, Male gender; the rate of female attendance at addiction clinics is very low, the Age range from 10 to 18Y, with average Intelligence quotient (IQ), Literate to understand the psychological instruments, agreed to sign the informed consent, follow-up cases who were abstinent for at least 2 weeks to avoid the effect of intoxication or withdrawal syndrome on the cognitive test assessment.

#### Exclusion criteria of the case group

The presence of significant physical illness, ‘which might interfere with cognitive task performance, the presence of co-morbid neurological illness to avoid their effects on the results, also intellectual disability to avoid their effects on the results, patients having neurocognitive disorders, patients diagnosed with attention-deficit/hyperactivity disorder (ADHD) according to DSM-5 criteria for diagnosis of the psychiatric disorder, and patients having intoxication or withdrawal syndrome.

#### Control group

Adolescents with no substance use disorder were matched with the patients based on demographic variables: age, gender, residence, educational level, and economic status, with no current psychiatric disorders, intellectual disability, neurological, or neurocognitive disorders.

### The following tools of assessment were used

#### Clinical assessment

##### A structured sheet created for research, including sociodemographic and clinical characteristics

Collection of information regarding age, residence, educational level, age at first use of the substance, number of relapses, frequency of substance consumption, and types of substances.

##### MINI-KID

MINI-KID is a brief, semi-structured diagnostic interview for children ages 4 to 17 that was created in collaboration with psychiatrists and physicians in the USA and Europe. The MINI-KID modules address a wide range of conditions, including eating disorders, pervasive developmental disorders, bipolar disorders, anxiety disorders, OCD, PTSD, alcoholism, drug addiction, tic disorders, ADHD, disruptive disorders, psychotic disorders, and eating disorders. The test looks for 24 DSM-IV and ICD-10 mental diseases as well as suicidality. Research from the past has indicated that the interview takes about half an hour to complete if the interviewer has sufficient training and a basic grasp of mental illnesses. It should be sufficient as a quick but reliable interview that may be used for both clinical and research goals, according to Sheehan et al. [11]. The Arabic translation was conducted by Kadri et al. [12].

#### Psychometric assessment

##### Assessment of IQ: (Stanford- Binet Intelligence Scale, 5th edition): was applied for the case and the control groups

It was used to make sure that the participant’s IQ fell within the average range. The test includes both verbal and nonverbal subtests and weighs five factors in total. The following five criteria are being tested:

• Knowledge.

• Quantitative reasoning.

• Visual-spatial processing.

• Working memory.

• Fluid reasoning.

[13]. Scores are reduced to a ratio known commonly as the intelligence quotient, or IQ. Scores of 90–109 are considered to be average [14].

##### Assessment of some aspects of executive functions by Trail Making Test (part A and part B): was applied for the case and the control groups

Numerous performance-based EF measures have been utilized for years with the adolescent subpopulation. These comprise tasks like the Trail Making Test [15]. TMT has an important spatial component that is connected to the right hemisphere function. The execution involves logical and sequential reasoning, which is why it is more associated with the left hemisphere function. Additionally, it assesses working memory, mental flexibility, inhibitory capacity, alternate-attentional ability, and anticipatory ability [16].

The subject is asked to link a series of markers while taking the test. A participant must make as many connections as possible on a page with the numbers (1–25) marked. Part B has both numbers and letters instead of only numbers. The participant is given a paper with circled numbers (1–13) and letters (A–L) and must make connections between them as rapidly as possible (e.g., 1-A-2-B-3-C, etc.) [17].

##### Assessment of quality of life using WHO QoL-BREF: was applied for the case and the control groups

The WHOQOL-BREF is a shortened version of the WHOQOL-100 scale. It is a generic questionnaire with twenty-six items. There are several possible responses to its questions, ranging from 1 (very dissatisfied/very poor) to 5 (very satisfied/very good). It has 4 domains: physical health (seven items), psychological health (six items), social relationships (three items), and environmental health (8 items), as well as items for general health and quality of life (QOL). 1. The physical health domain covers topics related to mobility, daily activities, functional ability, energy, discomfort, and sleep. (3), (4), (10), (15), (16), (17), and (18). 2. The psychological domain measurements cover self-image, unfavorable thoughts, favorable attitudes, self-esteem, mentality, learning capacity, memory focus, and mental status. (5, 6, 7, 11, 19, and 26). 3. The social relationships area of the test includes all aspects of interpersonal interactions, social support, and sexual life. These two queries (20) and 21. 4. Topics like transportation, noise, air pollution, and other general environmental challenges are included in the environmental health domain, together with financial resources, safety, health, and social services. It also offers chances to pick up new knowledge and abilities. (8), (9), (12), (13), (14), (23), (24), and (25) [18].

#### Laboratory assessment

Urine toxicology screen: Urine samples were collected in sterile containers and examined using the multidrug 1-step test.

### Sample size calculation

The calculated sample size of the study was 43 participants at a 5% level of significance and 80% power, using the following formula:

$$N = ~\left( {Z1 - \alpha /2 + Z1 - \beta } \right)~~\sigma 1*~\sigma 2~/~\delta ^{2}$$

$$Z1-\alpha /2 = 1.96$$

$$Z1-\beta = 0.842$$

$$\sigma = SD (10.5, 10.6)$$

δ = Expected difference to be detected in global composite among low and high-risk alcohol use (4.5) [19].

α = Level of acceptability of a false positive result (level of significance = 0.05).

β = Level of acceptability of a false negative result (0.20).

β = power (0.80).

The sample size was increased to 50 participants to increase the study power.

### Statistical analyses

The computer was fed with data, and IBM SPSS Corp.'s 2013. Version 22.0 of IBM SPSS Statistics for Windows. IBM Corp., Armonk, New York. Numbers and percentages were used to describe qualitative data. After confirming normality with the Kolmogrov-Smirnov test, quantitative data were presented using the median (minimum and maximum), mean, and standard deviation for parametric data. The (0.05) level was used to determine the significance of the obtained results. Chi-Square, Monte Carlo, and Fischer exact tests for the comparison of two or more groups were used for qualitative data. Quantitative data; parametric tests: Student t-tests were employed to compare two independent groups. Non-parametric tests: Mann–Whitney U test combined with Spearman's correlation were used to compare two independent groups. The strength and direction of a linear relationship between two non-normally distributed continuous variables or ordinal variables are assessed using Spearman's rank-order correlation.

## Results

### Sociodemographic characteristics of the sample

There was no significant difference in the sociodemographic characteristics of the studied groups. The mean age of the control group is 15.3 versus 15.26 years among cases. All studied groups are males, and the educational level of the studied groups is mostly secondary education 72% among control versus 70% among cases as shown in Table 1

**Table 1:** comparison of sociodemographic characteristics between the studied groups

	Cases n = 50	Control n = 50	Test of significance
Age/years			
mean ± SD	15.30 ± 1.46	15.26 ± 1.45	t = 0.137 p = 0.891
Residence			
Rural	42 (84)	40 (80)	χ2 = 0.271
Urban	8 (16)	10 (20)	p = 0.603
Educational level			
Preparatory	2 (4)	2 (4)	χ 2 = 0.054
Secondary	36 (72)	35 (70)	p = 0.973
University	12 (24	13 (26)	χ 2 = 0.054

t: Student t-test, χ2: Chi-Square test, SD: standard deviation

### Clinical characteristics of the case group

The mean age of onset was lower in the Poly substance group (15.5 ± 2.1) than in the Mono substance group (17.2 ± 1.0) and this difference was statistically significant. As regards the type of substances used among substance users. 86% of cases are polysubstance users and 14% are mono-substance users distributed as follows: 71.4% opioids and 28.6% cannabis as shown in Table 2.

**Table 2:** Distribution of studied cases according to clinical characteristics

	Mono-substance n = 7	Poly-substance n = 43	Test of significance
Age of onset/years	17.2 ± 1.0	15.5 ± 2.1	t = 2.08 p = 0.042*
Number of Relapses	1(14.3)	2(4.7)	FET = 0.99 P = 0.319
Frequency			
• Occasionally	1(14.3)	3(6.98)	MC = 1.60 P = 0.448
• Daily	6(85.7)	33(76.7)	
• Every other day	0	7(16.3)	
Types of Substances			
Opioids	5 (71.4)	39 (90.7)	χ^2^ = 2.12 P = 0.145
Cannabis	2 (28.6)	37 (86.1)	χ^2^ = 11.59 P = 0.0006*
Sedatives	0	9 (20.9)	χ^2^ = 1.78 P = 0.181
Amphetamine	0	8 (18.6)	χ^2^ = 1.55 P = 0.213
Tramadol	0	24 (55.8)	χ^2^ = 7.51 P = 0.006
Cocaine	0	0	

*t* Student t-test, *FET* Fischer exact test, *MC* Montecarlo test *χ*^*2*^ Chi-Square test

### TMT results in the cases and controls

There was a statistically significantly higher mean TMT A and TMT-B score among studied cases than the control group with a mean score of TMT-A 74.38 versus 63.2 among the control and for TMT-B; the mean score for the control is 97.22 versus 142.04 among cases as shown in Table 3.

**Table 3:** comparison of TMT A& B between studied groups

	Control n = 50	Cases n = 50	Test of significance
TMT-A	63.20 ± 14.97	74.38 ± 34.40	t = 2.11 p = 0.038*
TMT-B	97.22 ± 45.68	142.04 ± 58.68	t = 4.26 p < 0.001*

*TMT-A, B* trail making test A, B, *t* Student t-test, *statistically significant

### Quality of life results in the cases and controls

There was a statistically significant lower median score among cases than controls. The median score for overall and general health domain, Physical health domain, Psychological Health, Social relationship, Environmental health, and total score of quality of life is 76.5, 58.33, 50, 76.92, 70.59 & 60.78 for the control group versus 60, 41.18, 26.67, 50, 41.18, and 31.01 among cases group as shown in Table 4.

**Table 4:** comparison of quality of life scores between studied groups

Quality of life	Control n = 50	Cases n = 50	Test of significance
Overall & general health	75 (66.67–80)	60 (0–80)	z = 6.82 p < 0.001*
Physical health	58.33 (37.5–64.29)	41.18 (0–61.54)	z = 7.01 p < 0.001*
Psychological health	50 (15.38–59.26)	26.67 (0–50)	z = 7.13 p < 0.001*
Social relationship	76.92 (57.14–80)	50 (0–76.92)	z = 7.71 p < 0.001*
Environmental Health	70.59 (54.55–74.36)	41.18 (0–65.52)	z = 8.15 p < 0.001*
Total score	60.78 (42.03–66.1)	31.01 (0–56.52)	z = 7.98 p < 0.001*

Parameters described as median (min–max), Z: Mann Whitney U test, *statistically significant

### The correlation between TMT results and quality of life

**Fig. 1:**
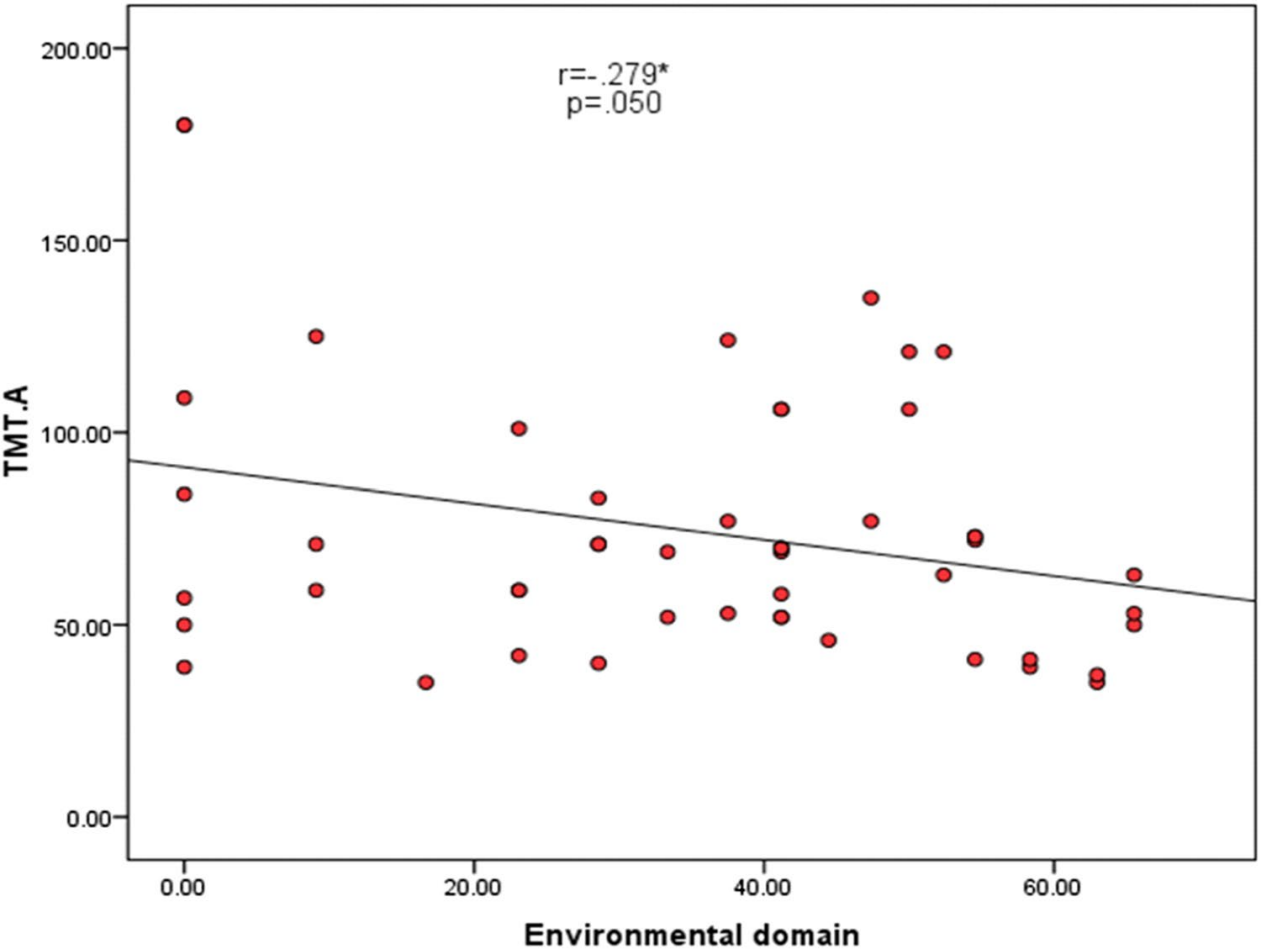
The relationship between TMT-A and the environmental domain of quality of life

**Fig. 2:**
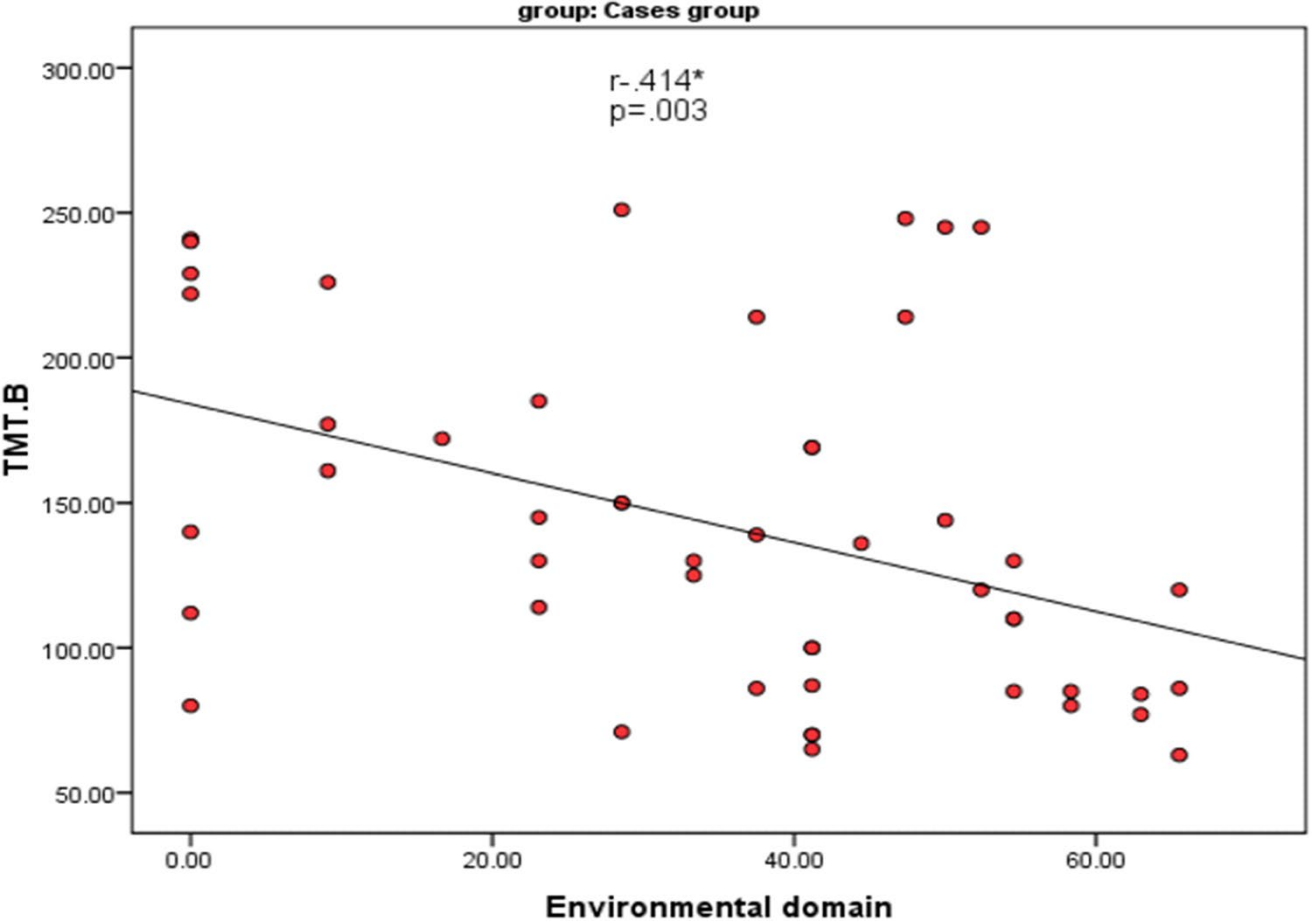
The relationship between TMT-B and the environmental domain of quality of life

There was a statistically significant negative correlation between TMT-A and the environmental domain of quality of life (r = − 0.279) and between TMT-B and the environmental domain of quality of life (r = − 0.414) as shown in Table 5, Figs. 1, and 2.

**Table 5:** correlation of quality of life scores with TMT among cases group

		TMT-A	TMT-B
Overall Quality of Life and General Health domain	R	− 0.226	− 0.259
	P	0.115	0.069
Physical health domain	R	− 0.016	− 0.063
	P	0.913	0.666
Psychological health domain	R	0.025	− 0.052
	P	0.862	0.721
Social health domain	R	− 0.241	− 0.148
	P	0.092	0.306
Environmental domain	R	− 0.279*^*^	− 0.414*^*^
	P	**0.050**	**0.003**
Total QOL score	R	− 0.201	− 0.257
	P	0.161	0.071

*QOL* quality of life, *r* Spearman correlation co-efficient, *statistically significant

## Discussion

Our research focused on the assessment of the executive functions and quality of life in adolescents with substance use disorders. According to the results of the current study; polysubstance users were found to be highly prevalent. 86% of the entire population had a poly-substance use disorder diagnosis; mono-substance users comprised 14% of the sample and were mostly opioid (71.4%) and cannabis (28.6%) users. These findings are consistent with those of Mahgoub and colleagues [20], who discovered that 12% of cases were positive for tramadol usage, 2% were positive for heroin use, and 43% of cases were positive for multiple drug use.

In the current study, we used Trail making test (TMT) to assess the executive function among the included cases. The current results showed that there was a statistically significant higher mean TMT-A and TMT-B score among studied cases than the control group with a mean score of TMT-A 74.38 versus 63.2 among controls and for TMT-B; the mean score for control is 97.22 versus 142.04 among cases. By Hagen and colleagues [21] it was shown that, with modest effect sizes, the control group considerably outperformed the SUD group on the Stroop Color Word (CW) variables word reading, color naming, and color/word naming. On the Stroop Interference task, there was no group difference. There was a significant group difference on TMT part A (d = 0.452, p = 0.028), but not on TMT part B (d = 0.086, p = 0.658).

According to Thoma and colleagues [22], who discovered a statistically significant difference between the executive functions (set-shifting) of addicts and those of a control group, the results of this investigation were consistent with their findings. Therefore, the executive function, cognitive flexibility, and capacity to transition between various mental activities in addicts were all lower than in the control group. This result is consistent with that of Mullan and colleagues [23] These investigations all showed that on tests measuring executive function and reaction inhibition, addicts underperformed compared to controls.

Our results were in line with those of a study conducted by Khalifa and colleagues [24]. In this study, 30 tramadol-dependent people were examined and compared with healthy controls regarding cognitive functions. They discovered that tramadol users suffer from memory and concentration problems. The findings of the current study were also consistent with the results of the studies demonstrating memory and cognitive impairments in heroin addicts as well as the findings of the studies demonstrating these impairments in abstinent opioid users [25, 26].

Another Egyptian study that compared 100 opioid-dependent Mansoura University students to matched controls based on how well they performed on the Wisconsin Card Sorting (WCST) and Trail Making tests revealed that opioid users often had executive dysfunction [20]. Contrarily, some additional research was unable to demonstrate the detrimental effects of heroin use on specific executive functions: This is consistent with Pau and his colleagues' research, which assessed how heroin affects executive functions and found that attention and mental flexibility were unaffected [27]. Examining various racial groups without considering their genetic differences could cause inconsistent results regarding executive dysfunction brought on by opiate addiction.

In the current study, all quality of life questions evaluating the following domains—general health and wellbeing, physical health, psychological health, social connections, and environment—found a statistically significant difference between the case and control groups. Ismail and colleagues' [28] findings involved two groups: controls (of the same age and gender) and patients (12–19 years old) with a diagnosis of drug misuse from Cairo, Egypt’s outpatient clinic for adolescents, Abbasiya Mental Health Hospital. All quality of life indicators included in this study—physical, cognitive, emotional, social, financial, and personal—showed a considerable difference between the two groups.

This supports the conclusions of Srivastava and Bhatia [29], who discovered a significant improvement in patients’ quality of life after abstaining from alcohol for 3 months. Before starting therapy, alcohol-dependent patients' physical, psychological, social, and environmental QoL scores were significantly lower than those of healthy controls. Measuring the quality of life in drug dependence is a crucial clinical indicator and a technique for identifying relapse risk factors. Scores were much worse in categories including mental health, economic well-being, social functioning, and productivity [30], maybe as a result of the multidimensional nature of SUD.

Birkeland and colleagues [31] discovered that the SUD group had a quality of life score that was relatively close to the average, which is opposite to our findings. The SUD group’s relatively high QoL may be due to the heightened stress in their personal and social lives, leading to considerable social support and family cohesion which was reflected in QoL scores.

According to the current study, TMT-A and the environmental domain of the WHOQOL BREF had a negative correlation (r = − 0.279 and r = − 0.414, respectively). Our results were consistent with those of Muller and colleagues [32] because the environment QoL domain was demonstrated to be the most sensitive to substance use and because its validity may be higher for those in active substance-using phases than for those in other groups. This conclusion may be explained by the indications of social inclusion provided by the environmental domain’s metrics, such as involvement in recreational activities and access to social care. Physical discomforts, such as pain and poor sleep, may make it more difficult for SUD sufferers to receive therapy, according to previous research [33].

## Limitations

Several limitations should be considered in interpreting the results of our study. First; The study’s findings are not generalizable because the patients were recruited from a single center and were small in number. Second; No subsequent reevaluation was performed on the cases after their initial outpatient clinic visit. Third; using only one test to evaluate the executive function which is a broader concept to be evaluated by only one test.

## Conclusions

According to the results of the current study, substance use disorders are a serious health issue for young people. In conclusion, this study was able to find the following: The Trail Making test scores, which represent some items of executive functions (set shifting, response inhibition, problem-solving, working memory, and mental flexibility), were found to differ statistically between adolescents with substance use disorders and healthy adolescents, with lower values being associated with the SUD group. On the other hand, the more affected executive functions are associated with lower quality of life in the case group.

## Data Availability

The data sets used and/or analyzed during the current study are available from the corresponding author upon reasonable request.

## Abbreviations

EFs: Executive functions
SUD: Substance use disorder
TMT: Trail Making test
AUD: Alcohol use disorder
QoL: Quality of life
DSM-5: Diagnostic and Statistical Manual-5 (DSM-5)
SCID-5: Structured clinical interview for DSM-5
HRQOL: Health-related quality of life
WHOQOL: World Health Organization Quality of Life
IQ: Intelligence quotient
Stroop CW: Stroop Color Word
WCST: Wisconsin card sorting test
